# Effects of neuromuscular blockade reversal on bispectral index and frontal electromyogram during steady-state desflurane anesthesia: a randomized trial

**DOI:** 10.1038/s41598-019-47047-1

**Published:** 2019-07-19

**Authors:** Doyeon Kim, Jin Hee Ahn, Hyunjoo Jung, Ka Young Choi, Ji Seon Jeong

**Affiliations:** 1 Department of Anesthesiology and Pain Medicine, Samsung Medical Center, Sungkyunkwan University School of Medicine, Seoul, Korea; 2 Department of Anaesthesiology and Pain Medicine, Kangbuk Samsung Hospital, Sungkyunkwan University School of Medicine, Seoul, Korea

**Keywords:** Outcomes research, Randomized controlled trials

## Abstract

The degree of neuromuscular blockade reversal may affect bispectral index (BIS) value. One possible reason is that the reverse of neuromuscular blockade affects electromyographic (EMG) signals of fascial muscle. Another reason is, the afferentation theory, the reverse of neuromuscular blockade relieves block signals generated in muscle stretch receptors from accessing the brain through afferent nerve pathways and induces arousal. Inaccurate BIS value may lead to overdose of drugs or the risk of intraoperative awareness. We compared changes in BIS and EMG values according to neuromuscular blockade reversal agents under steady-state desflurane anesthesia. A total of 65 patients were randomly allocated to receive either neostigmine 0.05 mg/kg, sugammadex 4 mg/kg, or pyridostigmine 0.25 mg/kg for neuromuscular blockade reversal under stable desflurane anesthesia, and 57 patients completed the study. The primary outcome was change in BIS and EMG values before and after administration of neuromuscular blockade reversal agents (between train-of-four [TOF] count 1–2 and TOF ratio 0.9). The change in BIS and EMG values before and after administration of neuromuscular blockade reversal agents were statistically different in each group (BIS: Neostigmine group, *P* < 0.001; Sugammadex group, *P* < 0.001; Pyridostigmine group, *P* = 0.001; EMG: Neostigmine group, *P* = 0.001; Sugammadex group, *P* < 0.001; Pyridostigmine group, *P* = 0.001; respectively). The BIS and EMG values had a positive correlation (*P* < 0.001). Our results demonstrate that the EMG and BIS values have increased after neuromuscular blockade reversal under desflurane anesthesia regardless of the type of neuromuscular blockade reversal agent. BIS should be applied carefully to measure of depth of anesthesia after neuromuscular blockade reversal.

## Introduction

Volatile anesthetics are commonly applied to induce and maintain general anesthesia. Minimum alveolar concentration (MAC) is one of the widely used indicator of preventing unexpected intraoperative awareness of volatile anesthetics^1,2^. Despite of its usefulness, the mechanism of immobility of MAC has been reported to be the spinal-motor neuron depression rather than analgesia and hypnosis^3–5^. Thus, the level of consciousness during surgery should be determined by the credible and objective indicators.

Bispectral index (BIS), since it was introduced in 1994^6^, has been widely used in various clinical situations including general anesthesia and sedation for procedures. BIS, represented by number, is the process of frontal electroencephalogram which demonstrating the awareness state and the degree of brain activity. It is the most widely used brain-function monitor, which is a noninvasive and simple method for assessing consciousness and depth of anesthesia by analyzing the frontal electroencephalogram (EEG) derived from the forehead^7,8^. Because BIS, range from 0 to 100, provides a patient’s anesthetic depth or conscious level in real time with objective values, it can avert adverse effects caused by excessive use of anesthetic agents or awakening during surgery due to light anesthesia. In addition, BIS affects the decision to decrease doses of anesthetic agents to prevent cardiovascular instability, including hypotension, without increasing the risk of intraoperative awareness in elderly or critically ill patients. However, despite its usefulness, the BIS as an indicator of level of consciousness should be interpreted with caution, since it is influenced by many factors including electrical interference, air vibration, and altered cerebral perfusion^9–12^.

Especially facial muscle activity results in inaccurate BIS during anesthesia because it involves electromyographic (EMG) over 30–40 Hz generated by muscle activity^13–16^. Previous research demonstrated that BIS values increased due to EMG contamination of EEG after administration of neostigmine or sugammadex as neuromuscular blockade reversal agents under propofol and remifentanil anesthesia with target-controlled infusion (TCI)^17,18^. On the other hand, according to afferentation theory, it produces muscle stretch or contraction, or directly stimulate muscle stretch receptors by reverse of neuromuscular blockade, will produce cerebral stimulation^19^. Therefore, reverse of neuromuscular blockade can affect EMG and BIS value. Contrary to intravenous (IV) anesthetics, volatile anesthetics cause muscle relaxation and enhance the effect of neuromuscular blockade by non-depolarizing muscle relaxants^20,21^. Because of these characteristics of volatile anesthetics, trends or changes in EMG during anesthesia may different from intravenous anesthesia. It is important to better understand the relationship between the degree of muscle relaxation and BIS value. Therefore, the aim of this study was to investigate the effect of neuromuscular blockade reversal agents on BIS and EMG values under steady-state desflurane anesthesia, a volatile agent.

## Methods

### Ethics

This prospective randomized study was approved by the Samsung Medical Center Institutional Review Board (IRB # 2017-12-096) and written informed consent was obtained from all patients in the trial. This was registered at the Clinical Trial Registry of Korea (https://cris.nih.go.kr, date of first registration: February/8^th^/2018, registration number: KCT 0002762) prior to recruitment of the first participant. All methods were performed in accordance with the relevant guidelines and regulations.

### Patients and randomization

Patients scheduled for elective laparoscopic cholecystectomy under desflurane anesthesia at our institution were assessed for eligibility and the patients who were suitable for this study were asked to participate in this trial (by DK). All patients participated in the study from March 2018 to May 2018. Inclusion criteria were American Society of Anesthesiologists (ASA) physical status I or II and age 19 to 70 years. Exclusion criteria were body mass index >30 kg/m^2^, pregnancy, severe liver disease, kidney function disorder, significant arrhythmia or cardiovascular disease, neuromuscular disease, and diseases for which the use of study drugs is contraindicated (ex: glaucoma, obstructive urinary tract disease, obstructive gastrointestinal tract disease, or severe ulcerative colitis).

Patients were assigned sequential numbers in order of enrollment and received their allocated treatment according to a computer-generated randomization schedule prepared before starting the study with the random permuted block method (1:1:1 allocation ratio and a block size of 3). Group allocation of the subjects was conducted by one of the authors (JHA) who was not involved in either study drug preparation or outcome assessment.

### Anesthesia protocol

Patients did not receive any premedication, and standard monitors, including electrocardiogram, pulse oximetry, heart rate, and noninvasive arterial blood pressure, were used in the operating theater. After each subject’s forehead was wiped with 70% alcohol and allowed to dry, a BIS quarto electrode was attached according to the manufacturer’s manual and connected to a BIS Vista monitor (2013; BISx Revision 1.15, BIS Engine 4.1). Thereafter, when the signal quality index (SQI) was confirmed to be more than 95%, BIS values were measured from the beginning of anesthesia induction to the end of anesthesia. Neuromuscular blockade was monitored by acceleromyography using the TOF-Watch SX^®^ (Organon Ltd, Dublin, Ireland) at the adductor pollicis. Each participant’s right arm was fixed to an arm board, and two pediatric electrodes were placed over the ulnar nerve near the wrist. After 5 s 50-Hz tetanic stimulus, TOF-Watch SX^®^ was calibrated using the automated CAL2 mode. Patients were preoxygenated with 100% O_2_ via facial mask, and anesthesia was induced with propofol 2 mg/kg and sevoflurane 8 vol%. Rocuronium 0.6 mg/kg was injected, and tracheal intubation was performed after confirming maximum neuromuscular blockade with twice Train-of-Four count (TOFC) 0. After intubation, sevoflurane was changed to desflurane 5–6 vol% and mechanical ventilation was adjusted to maintain end-tidal carbon dioxide level between 35 and 40 mmHg. The ulnar nerve was supramaximally stimulated with TOF mode every 15 seconds. When TOFC regained to 2 or higher intraoperatively, patients were given rocuronium 0.1 mg/kg. Once the skin suture has been finished, desflurane was adjusted to maintain a steady state with a BIS value in the range of 40–50 and was fixed through the study period.

After applying the surgical dressing and confirming TOFC 1–2, one of three neuromuscular blockade reversal agents was given by randomized sequence. The neuromuscular blockade reversal agents were prepared in a total of 10 mL and as the same appearance. Syringe contained neostigmine 0.05 mg/kg with glycopyrrolate 0.01 mg/kg in 0.9% saline for Neostigmine group, sugammadex 4 mg/kg in 0.9% saline for Sugammadex group, or pyridostigmine 0.25 mg/kg with glycopyrrolate 0.01 mg/kg in 0.9% saline for Pyridostigmine group. After confirming TOFR 0.9, desflurane was disconnected and the study was terminated. When the TOF ratio (TOFR) was not recovered to 0.9 by 20 minutes after administration of neuromuscular blockade reversal agents, the subjects received sugammadex 1–4 mg/kg to prevent adverse effects related to residual muscle relaxation according to degree of neuromuscular blockade reversal and these cases were discarded and not used in the analysis.

BIS, EMG values (Hz), and SQI were downloaded directly from the BIS-Vista monitor to a USB drive, and all neuromuscular results were stored in the personal computer by TOF-Watch SX^®^ computer interface. End-tidal desflurane (EtDes) and minimum alveolar concentration (MAC) based on age related iso MAC using anesthesia machine (Dräger Primus; Dräger Medical, Lübeck Germany) were recorded at the time TOFC 1–2 and TOFR 0.9 by a researcher who was blinded to the study. The primary outcome of this study was change in BIS and EMG values before and after administration of neuromuscular blockade reversal agents (between TOFC 1–2 and TOFR 0.9). The secondary outcome was the differences in BIS and EMG values depending on the degree of neuromuscular blockade (TOFC 1–2, TOFC 4, TOFR 0.5 and TOFR 0.9) among the three groups and time to regain TOFR 0.9.

### Statistical analysis

A power calculation was based on our unpublished pilot data and previous studies^17,18^. Aho *et al*. showed that changes in BIS had a mean of 7 and standard deviation (SD) of 7.5 after neostigmine administration^18^ and Vasella *et al*. demonstrated a mean (SD) change in BIS of 15 (7.5) in those treated with sugammadex^17^. In our pilot study, we found a mean of 8 and SD of 7.5 for differences in BIS after pyridostigmine administration. To detect statistical differences in changes of BIS values before and after administration of neuromuscular blockade reversal agents, one-way analysis of variance (ANOVA) test. With a power of 0.9 and an alpha error of 0.05, 20 patients were required in each group. Assuming a 10% dropout rate, we planned to enroll at least 66 patients (22 subjects for each group).

The changes in BIS and EMG values between before and after administration of neuromuscular blockade reversal agents were analyzed using a paired t-test or Wilcoxon signed-rank test as appropriate. Demographic and intraoperative data were analyzed using Pearson’s Chi-square tests or Fisher’s exact test for categorical variables and one-way analysis of variance (ANOVA) or Kruskal-Wallis test for continuous variables as appropriate. Normality of continuous variables was assessed by the Shapiro-Wilk test. Categorical variables were presented as numbers (percentage), and continuous variables were reported as means (SD) or medians (interquartile range, IQR). We used the generalized estimating equation (GEE) to compare BIS and EMG values of the three groups according to the degree of neuromuscular blockade. Bonferroni’s correction for post-hoc analysis was applied. Pearson’s correlation analysis was used to detect correlation coefficients between BIS or EMG values and the degree of neuromuscular blockade. Statistical analysis was performed using SPSS version 20 (IBM Inc., NY, USA), and *P* < 0.05 was considered statistically significant.

## Results

**Figure 1:**
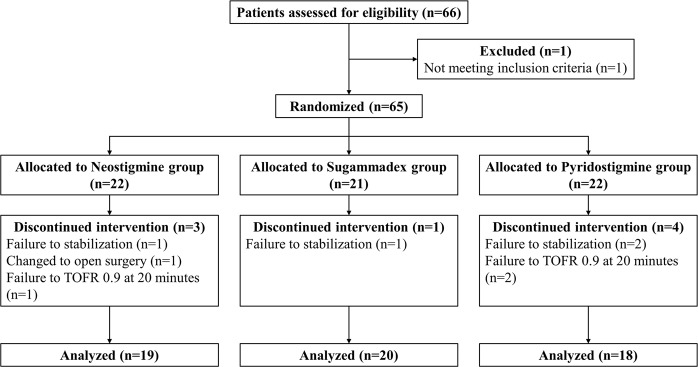
Consolidated Standards of Reporting Trials flow diagram.

A total of 66 patients were recruited for this study. Among these, one patient who did not meet the inclusion criteria was excluded from group allocation, and 65 patients were randomized into three groups (Neostigmine group, *n* = 22; Sugammadex group, *n* = 21; Pyridostigmine group, *n* = 22; respectively). Eight patients were excluded from the analysis after randomization because of failure to stabilize desflurane concentration in 4 cases, transition to open cholecystectomy in 1 case, and failure to regain TOFR 0.9 at 20 minutes in 3 cases. Finally, 57 subjects were analyzed (Neostigmine group, *n* = 19; Sugammadex group, *n* = 20; Pyridostigmine group, *n* = 18; respectively) (Fig. 1). Patient characteristics were not statistically different among the three groups (Table 1).

**Table 1:** Patient characteristics.

	Neostigmine (n = 19)	Sugammadex (n = 20)	Pyridostigmine (n = 18)
Age, yr	54.0 (47–62)	45.5 (33.8–56.8)	47.0 (37.5,56.5)
Sex, F/M	9/10	12/8	12/6
Height, cm	165.9 (157.7, 173.0)	162.8 (156.8–168.5)	160.95 (155.6–166.8)
Weight, kg	65.95 (55.9–73.0)	59.1 (53.5–66.7)	66.6 (56.8–73.9)
BMI, kg/m^2^	23.4 (22.1–26.3)	23.05 (20.8–24.6)	24.9 (22.3–27.63)
ASA class, I/II	9/10	14/6	10/7

Data are expressed as median (interquartile range) or number.

BMI, body mass index; ASA, American Society of Anesthesiologists.

**Figure 2:**
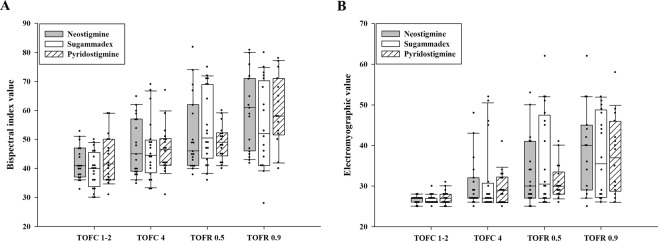
The bispectral index (**A**) and electromyographic value (**B**) depending on degree of neuromuscular blockade. Boxes represent the median with 25^th^/75^th^ percentile. Whiskers represent the minimum/maximum values, excluding outliers. Each dot represents a case. TOFC, train of four count; TOFR; train of four rate.

Figure 2 shows the BIS and EMG depending on degree of neuromuscular blockade. The median (IQR) in BIS value before and after administration of neuromuscular blockade reversal agents was 41 (37–47) and 61 (46–71) in Neostigmine group (median difference [MD], 18; 95% confidence interval [CI], 9 to 25; *P* < 0.001), 40 (34–46) and 52 (41–70) in Sugammadex group (MD, 14; 95% CI, 5 to 26; *P* < 0.001), and 42 (66–50) and 58 (52–71) in Pyridostigmine group (MD, 18; 95% CI, 10 to 25; *P* = 0.001). In addition, median (IQR) in EMG value before and after administration of neuromuscular blockade reversal agents was 27 (26–27) and 40 (29–45) in Neostigmine group (MD, 13; 95% CI, 5 to 17; *P* = 0.001), 26 (26–27) and 35 (27–49) in Sugammadex group (MD, 9; 95% CI, 1 to 19; *P* < 0.001), 27 (26–28), and 37 (29–46) in Pyridostigmine group (MD, 10; 95% CI, 3 to 15; *P* = 0.001). However, the difference in BIS and EMG values among the three groups was not statistically different (*P* = 0.797 and *P* = 0.781, respectively).

Intraoperative data are summarized in Table 2. At the time of TOFC 1–2, the baseline of this study, none of the subjects showed EMG over 35. The time to TOFR 0.9 was significantly shorter in Sugammadex group compared with Neostigmine group and Pyridostigmine group (*P* < 0.001). The BIS and EMG values depending on the degree of neuromuscular blockade was not significantly different among the three groups (*P* = 0.052 and *P* = 0.52, respectively). However, correlation coefficient between BIS value and degree of neuromuscular blockade was 0.528 in Neostigmine group, 0.453 in Sugammadex group, and 0.526 in Pyridostigmine group (*P* < 0.001, *P* < 0.001, and *P* < 0.001, respectively). In addition, correlation coefficient between EMG and degree of neuromuscular blockade reversal was 0.485 in Neostigmine group, 0.443 in Sugammadex group, and 0.581 in Pyridostigmine group (*P* < 0.001, *P* < 0.001, and *P* < 0.001, respectively). No patient reported any complications related to neuromuscular blockade reversal agents.

**Table 2:** Intraoperative data.

	Neostigmine (n = 19)	Sugammadex (n = 20)	Pyridostigmine (n = 18)	*P*-value
Total amount of rocuronium (mg)	45.42 (12.09)	41.3 (7.83)	45.00 (10.39)	0.402
Time to TOFR 0.9 (min)	11.26 (4.71)	3.05 (0.76)	14.22 (3.80)	<0.001
Duration of surgery (min)	53.63 (15.33)	52.50 (11.05)	56.33 (19.52)	0.740
Duration of anesthesia (min)	71.79 (17.88)	64.25 (10.75)	76.11 (19.02)	0.080
**At the time of TOFC 1–2**				
EtDes (vol%)	3.91 (0.81)	3.87 (0.53)	4.20 (0.43)	0.215
MAC	0.65 (0.13)	0.64 (0.09)	0.69 (0.09)	0.342
BIS value >60	0	0	0	—
EMG value >35	0	0	0	—
**At the time of TOFR 0.9**				
EtDes (vol%)	3.64 (0.99)	3.84 (0.64)	4.2 (0.67)	0.096
MAC	0.62 (0.16)	0.61 (0.13)	0.69 (0.12)	0.161
BIS value >60	10	9	8	0.853
EMG value >35	12	10	11	0.669

Data are expressed as mean (SD) or number.

TOFR, Train-of-Four ratio; TOFC, Train-of-Four count; EtDes, end-tidal desflurane; MAC, minimum alveolar concentration; BIS, bispectral index; EMG, electromyography.

## Discussion

Our results showed BIS and EMG values significantly increased after neuromuscular blockade reversal regardless of the type of reversal agents under steady-state desflurane anesthesia. The changes in both values after neuromuscular blockade reversal were not significantly different depending on the type of neuromuscular blockade reversal agents. In addition, BIS and EMG values have a positive correlation with the degree of neuromuscular blockade reversal regardless of the type of reversal agents.

The effect of anesthetics on intensity of muscle relaxation and central cholinergic activation associated with the use of anticholinergics might differ according to the type of anesthetic. Volatile anesthetics compared to IV anesthetics enhance the degree of neuromuscular blockade^22–24^. Bock *et al*. investigated the potency of rocuronium under steady-state anesthesia using various types of anesthetics and demonstrated that there was prominent synergism between the neuromuscular blockade effects of rocuronium and volatile agents^25^. The enhancing effect of volatile anesthetics is related to stimulation of antagonist affinity at the receptor site^20^ and the volatile anesthetics promote skeletal muscle relaxation^26^.

Volatile anesthetics with muscle relaxation effect may affect the EMG values and consequently affect the BIS value. However, the previous studies examined changes in BIS and EMG values under propofol and remifentanil anesthesia^13,17,18,27,28^, but the results are conflicting. Vasella *et al*. compared the effects of neuromuscular blockade and anticholinergics during propofol and remifentanil anesthesia by TCI^17^. They reported that administration of neostigmine as a neuromuscular blockade reversal agent (in contrast to administration of atracurium) significantly altered BIS during a steady-state level of IV anesthesia. They suggested that neostigmine changes the depth of IV anesthesia and may enhance recovery of consciousness. Conversely, Illman H *et al*. reported that depth of anesthesia represented by the BIS value and entropy were not affected by sugammadex administration^28^. They explained that the results related to rapid recovery of the neuromuscular function of sugammadex, which is not immediately reflected by BIS or entropy. Our results showed that BIS and EMG values significantly increased in desflurane anesthesia. Regardless of the type of neuromuscular blockade reversal agents, depending on the degree of neuromuscular blockade and BIS and EMG values are highly correlated. Two possible theory can explain why BIS and EMG levels are increased in patients who are reversed by neuromuscular blockade. One possible reason is that the reverse of neuromuscular blockade disturbs EMG signals, arousing from the muscles of the forehead, from falsely elevating BIS levels. The increased BIS value might be caused by high EMG activity which demonstrated electronic signal of muscle. Another reason is, the afferentation theory, the reverse of neuromuscular blockade relieves block signals generated in muscle stretch receptors from accessing the brain through afferent nerve pathways and induces arousal^19,28,29^. In this study, deafferentation can be caused by blockade of muscle spindles by the neuromuscular blockade and results in keeping of the increased BIS value. Unfortunately, our findings are difficult to distinguish between these two theories. Altogether, the possible effect of neuromuscular blockade on the quantitation of depth of anesthesia is still under debate. Therefore, it would be necessary to take a further study to distinguish the two theories, and BIS should be applied carefully to measure of depth of anesthesia after neuromuscular blockade reversal.

Generally, the BIS scale ranges from 100 (full awake EEG) to 0 (isoelectrical EEG, absence of brain activity)^30^, and a range of values between 40 and 60 is considered appropriate to prevent awareness during general anesthesia^31^. Because EEG results obtained by surface electrodes attached to a patient’s forehead, the recorded signal might consist of a neurological component (EEG) and a muscular component (EMG)^32^. EMG overlaps EEG (0–50 Hz) in the range of 30–300 Hz. Thus, it simulates EEG as a marker of consciousness (40–60 Hz)^33,34^, which can result in high BIS values. Dahaba *et al*. compared differences in neuromuscular blockade reversal agents including sugammadex and neostigmine in patients with high or low EMG values and reported that changes of BIS values were dependent on the existing muscle activity at the corresponding time^27^. However, in our study, high or low EMG values was not associated with elevation of BIS value. Moreover, high EMG values above 35 was identified in 0/57 (0%) patients at TOFC 1–2 before administration of neuromuscular blockade reversal agents, which was less than in other studies. This may be due to the muscle relaxation effect of volatile anesthetics. However, there was a tendency to increase both BIS and EMG values according to the degrees of neuromuscular blockade reversal during desflurane anesthesia.

Pyridostigmine is one of the most popular neuromuscular blockade reversal agents in Korea because it has fewer CNS and cholinergic effects compared with neostigmine^35,36^. However, as residual paralysis may occur despite the use of large amounts of pyridostigmine or neostigmine as neuromuscular blockade reversal agents for steroidal non-depolarizing agents^37^ and there were few researches which investigated the effect of pyridostigmine for neuromuscular blockade reversal. Careful use is required to avoid adverse consequences of residual paralysis and future study is needed to avoid adverse outcomes.

Adequate hypnosis during anesthesia is associated with unconsciousness and absence of postoperative recall of intraoperative events, and can be measured non-invasively via BIS^38^. The neuromuscular blockade has been known to be decoupled in terms of dynamic effect from the hypnosis and BIS. So, neuromuscular blockade has been essentially controlled separately from other parameters such as the BIS and Richmond Agitation-Sedation Scale, mean arterial pressure and cardiac output. However, our study showed that EMG and BIS increase by neuromuscular blockade reversal. Therefore, this should be considered in adequate hypnosis control because the BIS can vary depending on the degree of neuromuscular blockade. It is well known that there are differences in the reaction of cardiovascular, neurological function between volatile and IV anesthetics^39,40^. Volatile anesthetics are affected by the respiratory system and muscle relaxation than intravenous anesthetics^38^. Moreover, previous study investigated the effect of anesthetic type to EEG and showed that sevoflurane resulted more frequent incidence of excitement-disinhibition than other IV anesthetics including midazolam and propofol during sedation^41^. Therefore, BIS and EMG values due to reversal of NMBA in IV and volatile anesthetics may be different.

Our study has several limitations. First, there was no control group in this study. Ideally, a group of subjects who were not reversed would have been studied for comparison. It was not possible to establish a control group because it was considered unethical not to reverse neuromuscular blockade after use of muscle relaxant such as rocuronium. Second, patients receiving IV anesthesia did not compare. Because the effect of neuromuscular blockade reversal of propofol and remifentanil anesthesia was investigated in previous study^27^, we did not select patients under IV anesthesia as a study group. Further research is required to evaluate the effect of neuromuscular blockade reversal depending on the type of anesthetics. Finally, because the duration of laparoscopic cholecystectomy is usually less than 1 hour, a relatively small amount of muscle relaxant was administered compared to open abdominal surgery. Therefore, results may be different in prolonged surgery using a high dose of neuromuscular blockade agents. To compensate, TOFC was monitored during surgery and maintained in the range of 0–1.

In conclusion, our results demonstrate that the EMG and BIS values have increased after neuromuscular blockade reversal under steady-state desflurane anesthesia, regardless of the type of neuromuscular blockade reversal agent. However, it is difficult to determine whether the increased BIS after neuromuscular blockade reversal is related to increased EMG, and whether it is a true reflection of a changed arousal state. Therefore, BIS should be applied carefully to measure of depth of anesthesia after neuromuscular blockade reversal.

### Implication statement

Due to the muscle relaxation effect of volatile anesthetics, the electromyographic (EMG) artifacts may be different from intravenous anesthesia. We compared changes in bispectral index (BIS) and EMG values according to neuromuscular blockade reversal under steady-state volatile anesthesia. Our results demonstrate that the EMG and BIS values have increased after neuromuscular blockade reversal under desflurane anesthesia regardless of the type of neuromuscular blockade reversal agent.

## Data Availability

The authors intend to share, if requested by the journal, all the deidentified participant individual data, the initial protocol, and approval by the ethics committee. The data can be accessible by contacting the corresponding author.
